# Lemmel's Syndrome: A Rare Complication of Periampullary Diverticula

**DOI:** 10.7759/cureus.36236

**Published:** 2023-03-16

**Authors:** Arwa Battah, Iyad Farouji, Theodore R DaCosta, Nicholas D Luke, Dema Shamoon, Theodore DaCosta, Yatinder Bains

**Affiliations:** 1 Internal Medicine, Saint Michael's Medical Center, Newark, USA; 2 Cardiology, Saint Michael's Medical Center, Newark, USA; 3 Gastroenterology, Saint Michael's Medical Center, Newark, USA; 4 School of Medicine, St. George's University, True Blue, GRD; 5 Internal Medicine, New York Medical College at Saint Michael's Medical Center, Newark, USA

**Keywords:** periampullary diverticula, lemmel's syndrome, cholangitis, acute cholangitis, endoscopy, periampullary diverticulum, obstructive jaundice

## Abstract

Periampullary diverticula are outpouches in the mucosa in the duodenum surrounding the ampulla of Vater. Most cases of periampullary diverticuli are asymptomatic, but complications can arise, increasing a patient's mortality. Diagnosis of periampullary diverticuli is often incidental during endoscopy or imaging studies for abdominal pain. When a patient with periampullary diverticuli is symptomatic, imaging modalities such as CT scan and MRI can help make the diagnosis, but a side-viewing endoscope provides direct visualization of the diverticuli and also allows for the potential treatment of the disease. Lemmel’s syndrome is a complication of periampullary diverticuli where the diverticuli causes mechanical obstruction of the bile duct leading to obstructive jaundice without choledocholithiasis. These patients are at risk for further complications such as sepsis and perforation. Early diagnosis and treatment of these patients can help to prevent further complications from arising. We are presenting a case of Lemmel’s syndrome with obstructive jaundice from a periampullary diverticuli, further complicated by cholangitis without dilation of the biliary tree.

## Introduction

Gastrointestinal diverticula are outpouches in the mucosa involving all or part of the intestinal wall. Periampullary diverticula (PAD) are often asymptomatic and diagnosed incidentally during endoscopy [1]. Lemmel's syndrome occurs when a PAD causes mechanical obstruction of the common bile duct leading to obstructive jaundice. Most cases of periampullary diverticuli are asymptomatic [2]; however, complications such as Lemmel syndrome can occur. Lemmel's syndrome is often diagnosed using imaging modalities or under direct visualization. Treatment of patients with periampullary diverticuli is based on symptomatology and ranges from conservative management to diverticulectomy or sphincterotomy with or without stent placement [3]. Herein, we are presenting a case of Lemmel's syndrome causing obstructive jaundice and cholangitis without dilation of the biliary ducts.

This article was previously presented as a meeting abstract at the 2022 American College of Gastroenterology Annual Scientific Meeting on October 2022.

## Case presentation

A 58-year-old male presented to the emergency department with abdominal pain, nausea, and vomiting for two days duration prior to his presentation. He endorsed mid and upper abdominal discomfort overnight with non-bloody, non-bilious vomiting. He denied diarrhea, fever, or chills. His past medical history included two separate subdural hematoma evacuations a month prior to his current admission due to a head injury while snowboarding. The patient takes levetiracetam daily for post-traumatic epileptic episodes but denied headache and neck stiffness, and had no sick contacts at home or allergies. The patient stated he drinks one can of beer a week and never smoked. He did not have any endoscopies in the past. Upon admission, the patient developed a fever of 101.4°F, and tachycardia (pulse = 131 beats per minute). A physical exam revealed mid-epigastric tenderness without rebound, distention, guarding, or rigidity. Murphy’s and McBurney’s signs were also negative. Liver function tests revealed aspartate aminotransferase (AST) at 487 U/L (8-33 U/L), alanine aminotransferase (ALT) at 365 U/L (7-55 U/L), alkaline phosphatase at 155 IU/L (44-147 IU/L), and total bilirubin at 3.3 mg/dl (0.1-1.2 mg/dl). Glucose was 147 mg/dl on admission.

**Figure 1:**
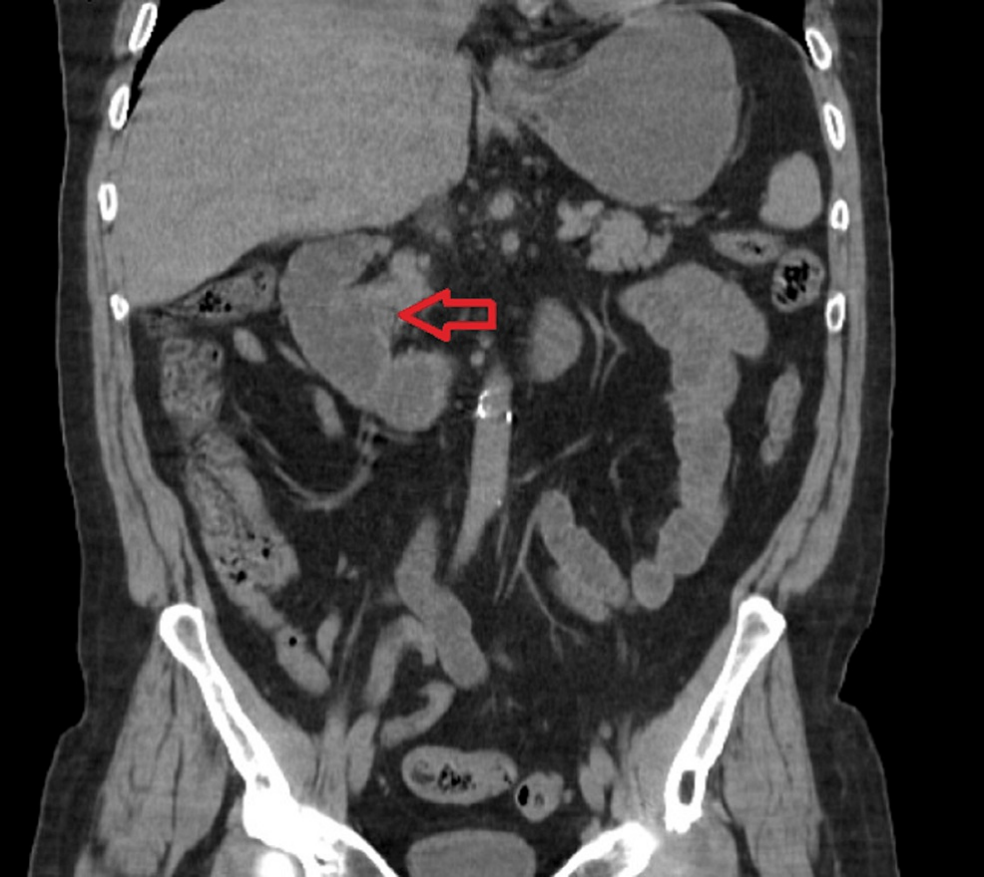
CT of the abdomen and pelvis without contrast showed a diverticulum at the ampulla of Vater (red arrow).

​A computed tomography (CT) scan of the abdomen and pelvis without contrast showed cholelithiasis with mild diffuse gallbladder wall thickening suggestive of acute cholecystitis with a diverticulum at the ampulla of Vater (Figure 1).

**Figure 2:**
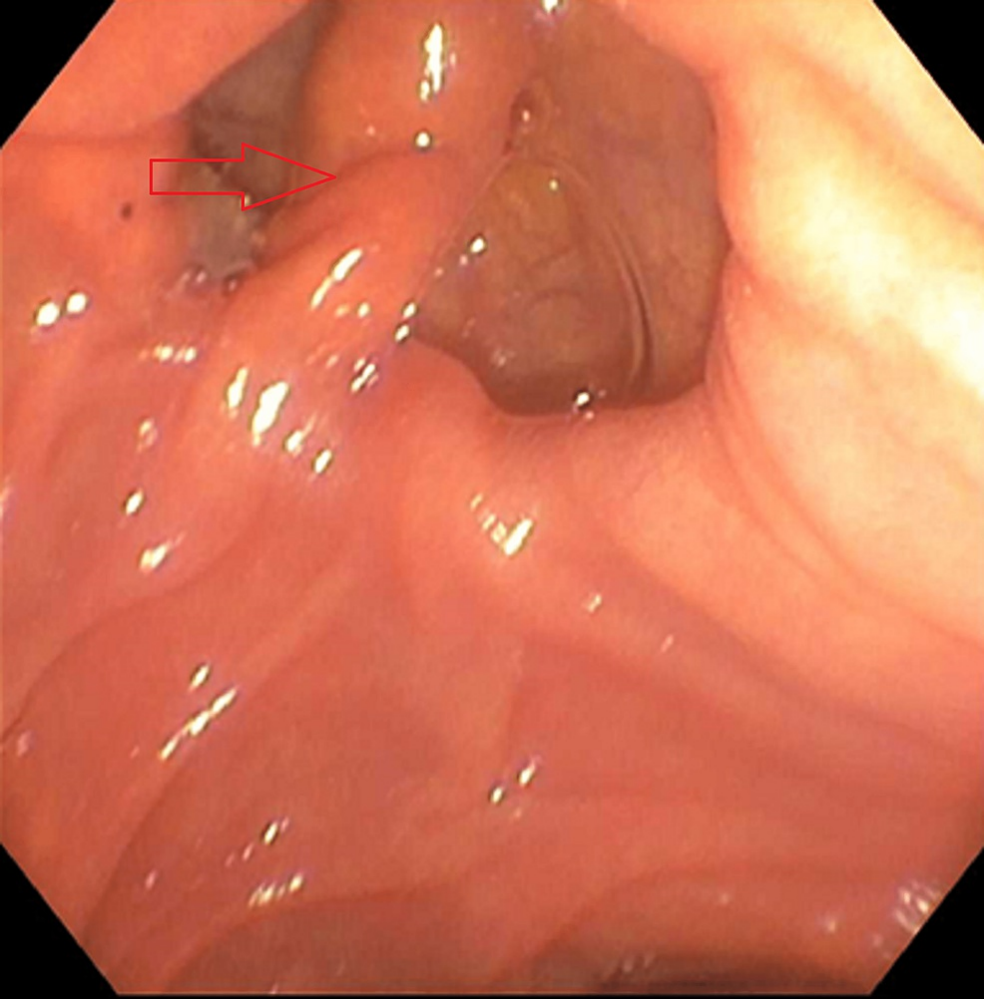
Upper endoscopy showing major papilla in the descending duodenum was located entirely within a large diverticulum.

**Figure 3:**
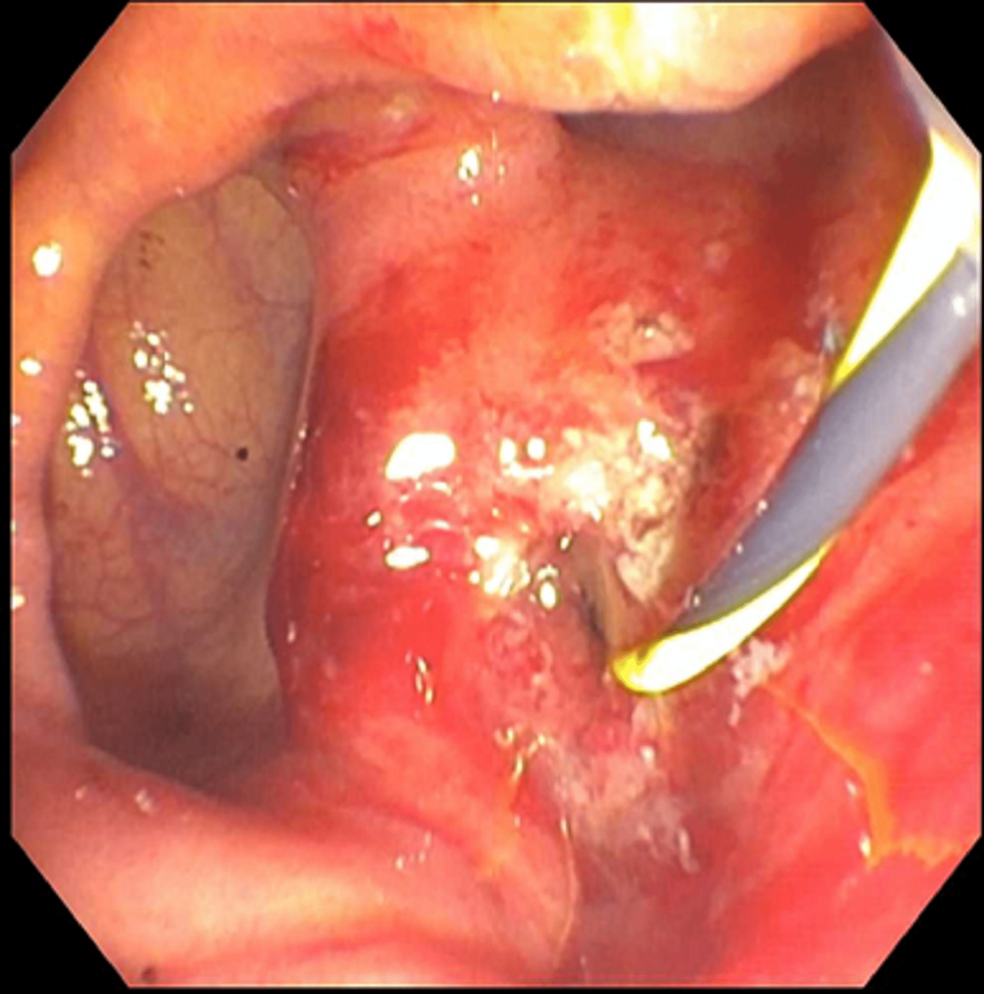
Endoscopic retrograde cholangiopancreatography (ERCP) revealed the major papilla in the descending duodenum was located entirely within a large diverticulum. A 0.035 x 260 cm straight Hydra Jagwire® was placed into the biliary tree, and one Advanix® Biliary 7 Fr by 7 cm plastic stent was placed into the common bile duct.

Upper endoscopy was grossly normal; however, the normal major papilla in the descending duodenum was located entirely within a large diverticulum (Figure 2). The pancreatic duct was deeply cannulated with a sphincterotome. A 0.035 inch x 260 cm straight Hydra Jagwire® (Boston Scientific, Marlborough, MA) was passed into the ventral pancreatic duct (Figure 3).

The biliary and pancreatic orifice pre-cut sphincterotomies were made with a sphincterotome using ERBE® electrocautery (ERBE, Marietta, GA). There was no post-sphincterotomy bleeding. One 4 French by 4 cm plastic stent was placed into the pancreatic duct. The bile duct was deeply cannulated with the sphincterotome and contrast was injected. The lower third of the main bile duct contained a tapered area in the distal common bile duct (CBD) that caused stenosis. No obvious stones were visualized. Placement of a 0.035 x 260 cm straight Hydra Jagwire® was placed into the biliary tree, and one Advanix® Biliary 7 Fr by 7 cm plastic stent (Boston Scientific, Marlborough, MA) was placed into the CBD. Bile flowed through the stent with no estimated blood loss. The patient was then returned to the ICU for ongoing care and remained nothing by mouth (NPO). Two days after the endoscopy, the patient developed disseminated intravascular coagulation (DIC) with sepsis secondary to ascending cholangitis and gram-negative bacteremia. Platelets dropped from 173 x 10^9^/L to 53 x 10^9^/L, international normalized ratio (INR) increased from 1.18 to 1.46, total bilirubin was 3.3 mg/dl, direct bilirubin was 1.94, ALT was 365 U/L, AST was 447 U/L, and alkaline phosphatase was 155 IU/L, and an abdomen CT showed cholelithiasis and diffuse gallbladder wall thickening that was concerning for cholecystitis. The patient also developed borderline iron deficiency due to anemia of chronic disease; iron saturation was 6.6%, with a total iron binding capacity of 165 mcg/dl (240-450 mcg/dl) and ferritin of 8 mcg/L (11-307 mcg/l). The patient's antiplatelet therapy and famotidine were discontinued and the patient was continued on IV meropenem to treat underlying sepsis/bacteremia.

## Discussion

Diverticula are outpouches of the gastrointestinal tract containing part or all of the intestinal wall [1]. Depending on the diagnostic modalities used, the incidence of diverticula in the duodenum ranges from 1% to 27% [1]. The most common diverticulum present in the duodenum is PAD, accounting for 70-75% [3]. The prevalence of PAD increases with age, rarely occurring below the age of 40 years, although this statistic may be underestimated due to the lack of testing in this age group [4]. PAD consists of extraluminal outpouching in the duodenum 2-3 cm surrounding the ampulla of Vater [2]. Most cases of PAD are asymptomatic and diagnosed incidentally during endoscopies, but a wide range of complications occur in 5% of the cases, such as bleeding, perforation, diverticulitis, pancreatitis, choledocholithiasis, cholangitis, and jaundice [5]. Lemmel's syndrome defined as hepatocholangiopancreatic disease leading to obstructive jaundice occurring in the absence of choledocholithiasis is another potential complication [6].

There are different theories regarding PAD and Lemmel's syndrome. One theory is chronic fibrosis secondary to diverticulitis, which can cause chronic inflammation with obstruction of the CBD [7]. Another potential mechanism is the sphincter of Oddi dysfunction, which may lead to mechanical obstruction [8]. Finally, indirect compression by an enterolith in the PAD can lead to obstruction of the CBD [9].

The diagnosis of Lemmel's syndrome is challenging. Many modalities can be used, including CT, MRI, and upper gastrointestinal endoscopy [10]. Though, a side-viewing endoscope during endoscopic retrograde cholangiopancreatography (ERCP) is the gold standard in diagnosing PAD and the origin of obstruction [11]. Imaging often shows thin-walled cavitary lesions located on the medial wall of the second portion of the duodenum with extrahepatic and intrahepatic biliary ductal dilatation [12]. These lesions often contain gas or fluid requiring a high index of suspicion from a radiologist, as they can be mistaken for pancreatic pseudocyst, pancreatic abscess, or even cystic neoplasm in the pancreatic head [12]. In our case, the patient presented with obstructive jaundice and cholangitis without dilatation of the biliary ducts, making this case more unique.

Treating patients with Lemmel’s syndrome varies based on symptomatology and pathophysiology. Conservative management is recommended in asymptomatic patients [13]. Patients presenting with cholangitis or obstructive jaundice, endoscopic extraction, extracorporeal shockwave lithotripsy, or surgery, such as a diverticulectomy or sphincterotomy with or without stent placement may be indicated [14].

## Conclusions

Lemmel’s syndrome is diagnosed when a periampullary diverticuli leads to obstructive jaundice in the absence of choledocholithiasis. Diverticuli is commonly found throughout the gastrointestinal tract, but it is less common in the duodenum. Of the diverticuli in the duodenum, periampullary diverticuli accounts for 70-75%. Most cases of periampullary diverticuli are asymptomatic and diagnosed incidentally, but complications of periampullary diverticuli can lead to complications of bleeding, perforation, pancreatitis, jaundice, and sepsis. Although there are multiple imaging modalities used to diagnose periampullary diverticuli, ERCP with a side-viewing scope is the gold standard. Treatment ranges from conservative management to surgery and often depends on symptomatology. Understanding the diagnoses of Lemmel’s syndrome can help provide insight into the disease and help physicians to diagnose and treat patients before complications occur.
